# Self-Reported Physical Activity is Not a Valid Method for Measuring Physical Activity in 15-Year-Old South African Boys and Girls

**DOI:** 10.3390/children5060071

**Published:** 2018-06-06

**Authors:** Makama Andries Monyeki, Sarah J. Moss, Han C.G. Kemper, Jos W.R. Twisk

**Affiliations:** 1 Physical Activity, Sport and Recreation Focus Area; Faculty of Health Sciences, North-West University, Potchefstroom 2520, South Africa; hanlie.moss@nwu.ac.za; 2 Amsterdam Public Health Research Institute, VU University Medical Centre, De Boelelaan 1085, 1081 HV Amsterdam, The Netherlands; hancgkemper@upcmail.nl (H.C.G.K.); jwr.twisk@vumc.nl (J.W.R.T.); 3 Department of Clinical Epidemiology and Biostatistics and EMGO-institute, Vrije Universiteit Medical Centre (VUmc), Vd Boechorststraat 7, 1081 BT Amsterdam, The Netherlands

**Keywords:** ActiHeart^®^, physical activity, adolescents, physical activity methods, physical activity and health longitudinal study

## Abstract

Physical activity plays an important role in the prevention of chronic lifestyle-related diseases. The development of valid instruments for the assessment of physical activity remains a challenge in field studies. The purpose of the present study was therefore to determine the level of agreement between physical activity objectively measured by the ActiHeart^®^ (Cambridge Neurotechnology Ltd, Cambridge, UK) device and subjectively reported physical activity by means of the International Physical Activity Questionnaire Short Form (IPAQ-SF) among adolescents attending schools in the Tlokwe Local Municipality, South Africa. A cross-sectional study design was used with a total of 63 boys and 45 girls aged 15 years who took part in the Physical Activity and Health Longitudinal Study (PHALS). Stature and weight were measured according to standard International Society for the Advancement of Kinanthropometry (ISAK) protocols. Objective physical activity (PA) was measured by a combined heart rate and accelerometer device (ActiHeart^®^) for seven consecutive days. Time spent in moderate-to-vigorous intensity physical activity (MVPA) was assessed. Subjective physical activity was assessed with the self-reported IPAQ-SF. Objective PA indicated that 93% of the participants were inactive and only 6% were highly active. The IPAQ-SF showed that 24% were inactive, with 57% active. A non-significant correlation (*r* = 0.11; *p* = 0.29) between the ActiHeart^®^ measure of activity energy expenditure (AEE) and total physical activity (IPAQ-SF) was observed. The Bland–Altman plot showed no agreement between the two measurement instruments and also a variation in the level of equivalence. When Cohen’s kappa (*κ*) was run to determine the agreement between the two measurement instruments for estimated physical activity, a poor agreement (κ = 0.011, *p* < 0.005) between the two was found. The poor level of agreement between the objective measure of physical activity (ActiHeart^®^) and the IPAQ-SF questionnaire should be interpreted cautiously. Future physical activity research using a combination of subjective and objective assessment methods in a large-scale cohort in adolescents is recommended.

## 1. Introduction

Studies indicate the important role of regular physical activity in the prevention of chronic diseases related to lifestyle [1,2]. Increased physical activity participation is highly recommended in order to decrease morbidity and mortality [3,4]. Since engagement in physical activity during childhood is found to track into adulthood [5] it is imperative to encourage children to sustain physically active lifestyles. To promote appropriate levels of physical activity in children, it is imperative to have precise measurement methods of physical activity in epidemiological studies [6].

Numerous methods in assessing physical activity are well documented [7,8,9,10]. It is ideal for physical activity instruments to capture all domains of physical activity, such as activity levels (i.e., low, moderate, and high intensities) during and after school, on week and weekend days, and during commuting, as well as sport and leisure activity and sedentary time [5,11,12,13,14]. Accurate assessments of physical activity are important for the advancement of research [15], especially in children [16]. Moreover, the selection of a method to determine physical activity must be based on careful consideration of the advantages and disadvantages of the instrument, as well as indications for application and evidence to support that the instrument is reliable and valid [6,17]. An objective measure of physical activity is ideal [18], but impossible to perform in large populations, and therefore alternative simple assessment methods of habitual physical activity are necessary [19]. Assessing physical activity is fraught with difficulties as it is multidimensional, and no single method can capture all subcomponents and domains in the activity of interest. Crude measures of physical activity may have led to inconsistent and false-negative results for the association between physical activity (or inactivity) and disease risk in epidemiological studies [20]. It has been alluded that the International Physical Activity Questionnaire (IPAQ), a physical activity questionnaire designed by a multinational working group as a common instrument for epidemiological studies, is suitable to use in adolescents from different settings [21,22]. Furthermore, IPAQ is the most commonly used subjective tool for evaluating physical activity because of its cost effectiveness and relative ease of use in large samples [23].

The differences between physical activity measure instruments (objective and subjective), result in the inability to compare physical activity levels between studies. Cross-validation between measurement instruments allows researchers to compare physical activity data across different methodologies, should the unit of measure be similar. Truthfully, validity should be reported as the degree of agreement between methods [24] because correlation coefficients in accordance with Schmidt and Steindorf’s [25] opinion may be misleading. A reliable questionnaire that overestimates physical activity to a large extent may correlate highly with an objective physical activity instrument [26,27]; these two measurements correlate but disagree. Such a questionnaire is considered valid to rank individuals (validity at the population level) but is not valid to measure physical activity with an absolute score (lack of validity at the individual level). Usually, self-reported instruments such as questionnaires show moderate to good reliability, but poor to moderate criterion validity (i.e., correlation coefficients of about *r* = 0.30 to 0.40), and absolute validity is often poor [14,22]. There seem to be sparse studies in South Africa which investigate the level of agreement between subjective and objective measures of physical activity. In addition, the current study may respond to a vital call made in the body of literature regarding the validation of physical activity questionnaires against criterion measures in studies on children [28,29,30,31]. The purpose of this study was therefore to determine the level of agreement between objectively measured physical activity by means of combined heart rate and accelerometry, ActiHeart^®^ (Cambridge Neurotechnology Ltd, Cambridge, UK) and the International Physical Activity Questionnaire Short Form (IPAQ-SF) among adolescents attending high schools in the Tlokwe Local Municipality of South Africa.

## 2. Methods

### 2.1. Participants

A cross-sectional study design was performed with a total of 63 boys and 45 girls aged 15 years who took part in the Physical Activity and Health Longitudinal Study (PHALS) [32]. Demographic information on age, gender, race, and socio-economic background (township or town) were assessed from the participants’ information. In addition, participants were asked to indicate their sport participation with the question: ‘Do you participate in sport?’. The question was answered by indicating “Yes” or “No”. Stature (cm) and weight (kg) were measured according to standard International Society for the Advancement of Kinanthropometry (ISAK) protocol [33].

Prior to the study, permission to conduct the measurements was granted by the District Manager of the Department of Education in Potchefstroom, South Africa. The protocol was approved by the Ethics Committee of the North-West University (Ethics number: NWU-0058-01-A1) of the Potchefstroom campus. Parents or guardians gave permission for their children to participate in the study, and participants provided informed assent for participation in the study. More details about the study are described elsewhere [32].

### 2.2. Objectively Measured Physical Activity

Physical activity was objectively measured using a combined heart rate and accelerometer device. The Actiheart^®^ instrument is a chest-mounted, light weight (10 g) instrument that uses synchronized heart rate (HR) and piezoelectric accelerometery data. This signal is converted to beats per minute (BPM) and written to the memory at the end of each epoch. The measurable range of HR in the manufacturer specification is 31–350 beat per minute, after which it was clipped onto two electrocardiography (ECG) electrodes on the left side of the chest according to the manufacturer’s instructions and recommendations detailed elsewhere [34]. Participants were told to wear the Actiheart^®^ device for 24 h for seven consecutive days. Activity data were recorded in 60-s epochs. The participants were instructed to carry on with their habitual lifestyle, keep the monitor on at all times (when awake and asleep), and only remove it when bathing, swimming, or partaking in high-impact sports (like rugby). Objective and subjective data were collected from August to October of 2011 during regular school term and weekend days. Data from the ActiHeart^®^ device were downloaded with commercial software (Version 2. 132, Cambridge Neurotechnology Ltd. Cambridge, UK). The software also recovered missing data by interpolating missing and noisy heart rates. The data were also trimmed to remove data of wear periods longer than seven days. The software helped in determining wear time and no-wear time as per the following categories: Actiheart^®^ software ‘OK’, ‘revered’, ‘interpolated’, ‘lost’, or ‘not worn’. Only accelerometer counts recorded under the classification ‘OK’, ‘recovered’ or ‘interpolated’ were used for calculating time spent in physical activity; this showed that Actiheart^®^ was detecting a signal, suggesting wear time. Recordings classified as ‘lost’ and ‘not worn’ were excluded from the analyses. Data from adolescents who wore the device for a minimum of four days (one of which was a weekend day) and for at least ten hours per day were included in the analysis.

Physical activity level (PAL) was calculated by dividing total energy expenditure (TEE) by estimated resting metabolic rate (RMR). The PAL cut-off points as described by the United Nations Food and Agriculture Organization (2001) are as follows: Sedentary (<1.40); light (1.40–1.60); moderate (1.61–1.99); heavy (2.00–2.40) and extremely heavy (>2.40). Heavy and extremely heavy physical activity scores were considered as ‘high’ [17]. The MET cut-off points which describe the intensity level of the physical activity were: Sedentary (<1.5 METs); light (1.5–3 METs); and moderate-to-vigorous (>3.0 METs) [34]. The CPM activity cut-offs for children aged 6–17 years were: sedentary (0–149 CPM); light (150–499 CPM); moderate (500–3999 CPM); vigorous (4000–7599 CPM) and very vigorous (>7600 CPM) [34]. Physical inactivity was described as performing insufficient amounts of physical activity, that is, not meeting specified physical activity guidelines [34]. The ActiHeart^®^ has been validated against doubly labeled water (DLW) in adults (*r* = 0.53, *r*^2^ = 0.29, *p* < 0.05) and adolescents (*r* = 0.23, *r*^2^ = 0.05, *p* = 0.36) [34].

### 2.3. Measurement of Subjective Physical Activity

Subjective PA was assessed using the IPAQ-SF [35,36,37], which was reported to be a valid and reliable tool for assessing PA [37]. The IPAQ-SF is considered suitable for use by adolescents in different settings [38] and the short form consists of seven items which identify the frequency and time spent in walking and engaging in other moderate-to-vigorous intensity PA during the seven days prior to administering the questionnaire. Only physical activity sessions that lasted ten minutes or more were analyzed with the IPAQ-SF. The IPAQ-SF also elicits information about time spent sitting, which is used as an indicator of sedentary time/behavior. The IPAQ-SF questionnaire was assessed after the ActiHeart^®^ was worn for seven consecutive days in order to be aligned with the objective physical activity recorded. To determine the intensity of the physical activity the METs were determined. Physical activity was classified into four categories in accordance with IPAQ Research Committee [37] classifications, namely: Low activity (METs < 3), moderate activity (METs 3–6), and vigorous activity (METs > 6). The debate and inconclusive agreement [38,39] on the use of the three or four METs in children may have affected the results of IPAQ-SF questionnaire. As such, we chose the use of METs in the current study based on the current physical activity (PA) guidelines for children and adolescents also using the METs to define moderate and vigorous activity [40]. Total physical activity (TPA) was calculated by adding all physical activity categories scores performed in seven days of the week.

### 2.4. Statistical Analyses

**Figure 1:**
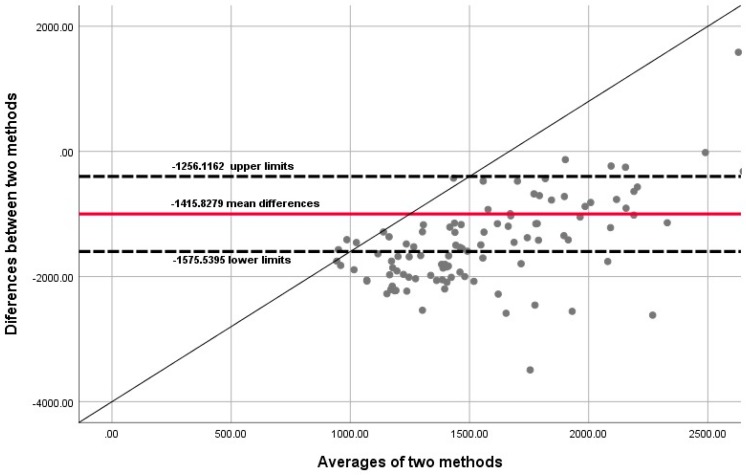
A Bland–Altman plot of the differences between the average of the objective activity energy expenditure (AEE) and total physical activity (PA) using the International Physical Activity Questionnaire Short Form (IPAQ-SF). The solid line is the average of the mean difference and the dotted lines are the 95% limits of agreement (inter-method difference is 1.96 standard deviation (SD) of the differences; upper and lower limits) between the AEE and the IPAQ-SF.

Statistical analyses were performed with the use of IBM SPSS (IBM Corp. Released 2017. IBM SPSS Statistics for Windows, Version 25.0. Armonk, NY: IBM Corp.) which is available from the North-West University network systems. Descriptive statistics (mean, min, maximum, standard deviation) and frequencies were determined. The Mann–Whitney U test was performed to determine the significant differences between males and females. The Bland–Altman method for continuous data and kappa (*κ*) for categorical data between ActiHeart^®^ and IPAQ-SF were applied. Cohen’s κ was calculated to measure the magnitude of agreement between objective physical activity measures determined with the ActiHeart^®^ and subjective physical activity with the IPAQ-SF. Spearman rank correlation coefficients (*r*) were calculated to determine the association between ActiHeart^®^ and the IPAQ-SF. Dixon and Pechmann [41], as well as Welk [42] describe the equivalence test as a technique that is used to examine measurement agreement (in the case of the current study it would be ActiHeart^®^ vs. IPAQ-SF). The approach implies that the null hypothesis states that the two methods are equal. Should the null hypothesis be accepted, the suggestion is that there is “no evidence of a difference”; however, Hauck and Anderson [43] argue that this “does not necessarily imply that there is evidence of equivalence”. As such, in equivalence tests, the null hypothesis is flipped to specify a difference between two means, thereby allowing a direct test of equivalence [44]. In our analysis, a 95% equivalence test (i.e., α = 5%) would help to conclude that ActiHeart^®^ and IPAQ-SF are considered significantly equivalent to each other if a 90% of confidence interval (CI) for the mean of the ActiHeart^®^ method falls into a proposed equivalence zone (i.e., ±10% of the mean) of the IPAQ-SF. Since the PA data of both subjective (IPAQ-SF) and objective (ActiHeart^®^) measurements were not normally distributed, the equivalence test was calculated by the use of a one-sample *t*-test and the non-parametric Wilcoxon signed-rank test to examine respectively the differences and the level of agreement between the differences between the two methods and the mean of the two methods. The differences between the averages of subjective (IPAQ-SF) (*y*-axis) and objective (ActiHeart^®^) methods were plotted against the average (*x*-axis) of the objective and subjective methods in the Blant–Altman graph (Figure 1). Significance was set at a level of *p* ≤ 0.05.

## 3. Results

The male participants were significantly taller and heavier (Table 1) as compared to their female counterparts. Data from ActiHeart^®^ show a significant gender difference for activity energy expenditure (AEE) in favor of the girls. With regard to IPAQ-SF, though not significant, boys showed higher mean values as compared to girls with respect to total physical activity.

Out of 108 participants, 90 (83%) adolescents participated in school sport, with 18 (17%) not taking part in any school sports. Male participants 50 (78%) participated less in sport compared to their female counterparts (*n* = 40; 91%). A total of 174 adolescents were issued with an Actiheart^®^, of which 108 (62%) successfully adhered to wear time and 66 (38%) did not comply with wear time or presented with incomplete IPAQ-SF information. No bias was observed between participants with complete data sets and those with incomplete data sets.

Data on the intensity of the physical activity from the ActiHeart^®^ classified 93% of the adolescents as low active, with only 1% moderately active and 6% vigorously active (Table 2).

The data from the IPAQ-SF, in contrast with the ActiHeart^®^ data, classified physical activity participation as 24% low active, 19% moderately active, and 57% highly active, with a Cohen’s κ of 0.011. Based on the guidelines from Altman [45], and adapted from Landis and Koch [46], a *κ* of 0.011 represents a poor strength of agreement. Furthermore, since *p* = 0.068 (which actually means *p* < 0.0005), our *κ* coefficient is statistically on the borderline of being significantly different from zero. When the Pearson Chi-square test was used to determine the observed distribution, a value of 4.88 with a *p*-value of 0.30 was found.

Results from the Spearman correlation coefficients indicated a significant positive correlation (*r* = 0.11; *p* = 0.29) between the ActiHeart^®^ measure of AEE and total physical activity (IPAQ-SF). However, the one *t* test of nonparametric technique revealed that the mean differences between the mean values of the two methods were statistically significant (*t* = −17.581, mean = −1415.8279; *p* = 0.001).

The measurement of agreement between the two physical activity measuring methods, assessed using a Bland–Altman plot, are shown in Figure 1. The Bland–Altman plots showed the differences between the two methods and the mean of the two methods to have poor limits of 95% confidence interval (CI) agreement (mean difference of −1415.8279; 95% CI −1575.5395 to −1256.1162; inter-method difference 1.96 SD of the differences). The mean differences between the two methods were scattered with no linearity along the liner line. The low percentage of vigorous PA as recorded with the Actiheart^®^ may be the reason for a non-linear association with relatively high values determined by IPAQ-SF. The Wilcoxon tests for the two one-sided tests used to test equivalence between the differences of subjective and objective PA are presented in Table 3. The probability level for equivalence test is less than the designated value of α (*p* < 0.05) (*Z* = −8.63; *p* < 0.001); we can reject the null hypothesis and conclude that the medians are equivalent for an equivalence margin of 1. Thus, the test required no assumption that the differences are normal. The results, therefore, show considerable variations in the individual differences between IPAQ-SF and Actiheart^®^, with yielded non-equivalent overestimation by IPAQ-SF self-reporting, as demonstrated by 57% of individuals being reported in the highly active category. Therefore, no agreement was found between the two measurement instruments.

## 4. Discussion

The purpose of the study was to determine the level of agreement between objectively measured physical activity (ActiHeart^®^) and the subjective measurement of physical activity with the IPAQ-SF among adolescents attending high schools in the Tlokwe Local Municipality of South Africa. The results show no significant level of agreement between ActiHeart^®^ and the IPAQ-SF in the assessment of physical activity. Additionally, there is no significant level of equivalence between the two methods for assessing physical activity. The reason for the low level of agreement observed in the study may be explained in part by the greater level of variability that was apparent for self-reported measures compared to objective measures.

A low non-significant positive correlation (*r* = 0.11; *p* = 0.29) between AEE (ActiHeart^®^) and total IPAQ-SF was found. The observed findings were somewhat similar to a study on primary school girls in Cape Town done by Mciza et al. [5] with the use of physical activity questionnaire (PAQ) and ACTIVITYGRAM, whereby in their study a significant positive association (*p* = 0.19; *p* < 0.001 and *p* = 0.26; *p* < 0.001 respectively) between PAQ-SF and intensity activities by ACTIVITYGRAM was reported. Furthermore, the observed low correlation was congruent with the findings from a review study by Helmerhorst et al. [10]. A similar correlation coefficient between ActiHeart^®^ and IPAQ-SF was reported in 24 boys and six girls aged between 16 and 20 in an Italian high school [47]. The lack of equivalence between the mean differences of ActiHeart and IPAQ-SF is somewhat incongruent with a study by Kim et al. [29] which compared the validity of Actigraph 2-regression models (2RM) and 1-regression models (1RM) for estimation of energy expenditure. It was revealed that none of the two 2RMs and four 1RMs were significantly equivalent to the indirect calorimetry method for both overall group comparisons and activity-specific comparison (with the exception of one activity with the Treuth 1 regression model (TH1RM)).

The lack of agreement between ActiHeart^®^ and the IPAQ-SF in our current study may be attributed to the well-known over-reporting of physical activity, which is mostly characterized by the problem of systematically recalling the exact activities performed in the previous seven days [13]. Additionally, there were coding errors with misclassification of intensity, duration, and frequency of PA bouts [47]. Vadone et al. [47] alluded that even a small variation in the expression and interpretation of PA recommendations has an enormous impact on the interpretation of objectively-derived estimates of physical activity. Assah and colleagues [48] report that a high prevalence of labor-intensive work involving activities such as digging, lifting, and load-carrying in rural dwellers reduced the amount of inter-individual variance in physical activity energy expenditure (PAEE) captured by the ActiHeart^®^ device, ultimately affecting the accuracy of the estimation for the more highly active subsample. Another reason for the observed of lack of agreement in the present study may be that participants reached a reporting threshold of a high level for physical activity scores (53%) with the IPAQ-SF as compared to ActiHeart (6%). While the types of activities performed by both township and urban adolescent learners would be significantly different compared with the types of sporting activities engaged in by the current sample, a high prevalence of different types of extracurricular sport and physical activity participation for many individuals in our sample (reported by IPAQ-SF) may also have reduced the degree of interindividual variability in PAEE. In establishing adolescent-specific PAEE prediction equations, particular attention should be afforded to accounting for the variation in activity levels and range of physical activities typical of the day-to-day behavioral patterns exhibited by this population. The equations used by the ActiHeart^®^ to estimate energy expenditure (EE) in adolescents were derived using data from 39 children aged 12–13 years during a treadmill protocol [49]. Additionally, this may be another reason for the lack of agreement between the two instruments since activities used during laboratory differ from activities performed in free-living settings [50,51,52], especially in the South African context. There exists a need to develop adolescent-specific prediction equations and validation of ActiHeart^®^ against a criterion reference standard with a population-specific cut-point [51] since inconsistency in the cut-points exists [8,44]. Hence, there is need for more comparative studies of the PA with the IPAQ-SF and ActiHeart^®^ for school-going adolescents [51], as some authors question the ability of the ActiHeart^®^ to accurately measure AEE in free-living adolescents [49,51]. Developmental differences between children, adolescents, and adults, in terms of metabolic costs and movement economy [53], impact the aerobic demand of activity for each age group. More specifically, biomechanical differences in walking and running between children, adolescents, and adults (e.g., faster stride rates, a higher body surface to body mass ratio, and greater muscle coactivation of the leg muscles), make children less economical compared with adolescents [54] and adolescents less economical in contrast to adults [55]. In spite of the advantages of objective measures like ActiHeart^®^, Barreira and colleagues [8] caution us about the inappropriate of this device for quantification of activities other than walking and running. For example, on studying the Actiheart monitor and other comparable measures, Actiheart^®^ underestimated energy expenditure for only one workload (jogging at 9.6 km/h) [8]. In a 2014 review by Hills and co-workers, the use of combined methods for assessing physical activity is highly recommended [13], since the use of subjective measures of PA have the potential to provide rich descriptive data, while objective instruments provide true reflection of the activity.

### Limitations

To our knowledge this is the first study to determine the level of agreement between the ActiHeart^®^ device and the IPAQ-SF in 15-year-old South African boys and girls. A lack of age appropriate group-based prediction equations may have limited the validity of the ActiHeart^®^ device for assessing school-going AEE in adolescents [10]. The absence of conducting the calibration step test during the set-up of the device might have influenced the results obtained and should in future be part of the standard protocol. The adolescents’ PA could have been influenced by the wearing of the ActiHeart^®^ device in the sense that they could have been more conscious of their own physical activity levels [52].

The scales of the outcome scores between ActiHeart and the IPAQ-SF are not the same since physical activity with IPAQ-SF is based on activity recall that assesses the individual ability to keep track of time, while physical activity with ActiHeart is based on the real-time physical activity that is performed. These factors may have contributed to lack of agreement between the two instruments. The IPAQ-SF sought information on the frequency and time spent in walking and engaging in other forms of moderate-to-vigorous intensity PA as well as time spent sitting, which is used as an indicator of inactivity on the one hand. The absence of the regression models in the analyses of the study (as done by Kim et al. [29]) may limit result comparison, of which further studies should make use of all different models of analyses to determine the level of agreement between physical activity measuring instruments. The reliance of adolescents to tell us when they last performed physical activity may have led to measured resting values being higher than expected, and as such might have had potential to affect activity-related METs values since an elevated resting MET value would result in lower activity METs [38]. As such, reliance of adolescents to provide information about PA is a limitation of this study. Additionally, caution is needed in the interpretation of a subjective measure like IPAQ-SF, since it has been reported that the questionnaire lacks the precision needed to detect physical activity on a day-to-day basis [12,56], which may have contributed to the low level of agreement between the two measurement instruments.

## 5. Conclusions

Our results showed substantial differences in the prevalence estimates between subjective measured physical activity compared to objective criterion measures. ActiHeart^®^ indicated that almost all the adolescents in the study were in the low physical activity category, while physical activities by questionnaire showed that 43% adolescents participated in low to moderate activity, with 57% participating in high-intensity physical activity. A poor level of agreement was found between the objective measure of physical activity by ActiHeart^®^ and subjective IPAQ-SF methods. Thus, the use of a combination of subjective and objective physical activity assessment methods in large-scale adolescent cohorts is recommended for future physical activity research.

## Figures and Tables

**Table 1:** Descriptive information (min, max, mean, standard deviation (SD) and *p*-values) of the participants.

		Min	Max	Mean	SD	*p*-Value of Gender
Stature (m)	Female	142.0	186.0	157.5	0.07	<0.001
	Male	155.0	191.0	170.0	0.08	
Body Mass (kg)	Female	33.8	93.0	55.22	11.08	0.04
	Male	38.1	105.0	60.47	13.72	
Body Mass Index (kg/m^2^)	Female	15	40	22.20	4.58	0.05
	Male	14.9	34.0	20.56	3.63	
AEE (kCal)	Female	258	1533	584.77	215.37	<0.003
	Male	181	878	462.15	153.30	
TPA (MET.min/week)	Female	16.0	1977.0	704.95	561.94	0.06
	Male	8.0	2488.5	941.49	639.45	

AEE (kCal) = activity energy expenditure (derived from objective measure); TPA = total physical activity (subjectively measured physical activity derived from the four PA categories); MET = Metabolic Equivalent.

**Table 2:** Percentages and Cohen’s kappa (*κ*) of physical activity intensity as assessed by ActiHeart^®^ and IPAQ-SF for different MET levels.

PA Level	PAL Categories (ActiHeart^®^)			IPAQ-SF	
	*n*		%	*n*	%
Low PA	100		93	26	24
Moderate PA	1		1	21	19
Vigorous PA	7		6	61	57
Total PA	108		100	108	100
***Symmetric Measures***					
		Value	Asymp. Std. Error ^a^	Approx T ^b^	Approx. Sig.
Measure of Agreement	κ	0.011	0.008	1.828	0.068
Number of Valid Cases		108			

^a^ Not assuming the null hypothesis. ^b^ Using the asymptotic standard error assuming the null hypothesis; MET = Metabolic Equivalent; *n* = number; % = percentage; PAL = physical activity level; PA = physical activity; IPAQ-SF = international physical activity questionnaire short form; Asymp. Std: Error = Asymptotic Standard Error; Approx T = Approximation Testing; Approx. Sig. = Approximation Significance.

**Table 3:** Equivalency analysis examining whether estimates are equivalent at the group level.

		*N*	Mean Rank	Sum of Ranks	
Averages of two methods – Differences between two methods	Negative Ranks	2 a	56.00	112.00	
	Positive Ranks	105 b	53.96	5666.00	
	Ties	0 c			
	Total	108			
					Averages of two methods – Differences between two methods (i)
				*Z*	−8.63 (ii)
				Probability level	0.001

a. Averages of two methods < Differences between two methods; b. Averages of two methods > Differences between two methods; c. Averages of two methods = Differences between two methods; i = Wilcoxon Signed Ranks Test; ii = Based on negative ranks.
